# Substantial heritable variation for susceptibility to *Dothistroma septosporum* within populations of native British Scots pine (*Pinus sylvestris*)

**DOI:** 10.1111/ppa.12528

**Published:** 2016-04-04

**Authors:** A. Perry, W. Wachowiak, A. V. Brown, R. A. Ennos, J. E. Cottrell, S. Cavers

**Affiliations:** ^1^ Centre for Ecology and HydrologyBush EstatePenicuikMidlothianEH26 0QBUK; ^2^ Institute of DendrologyPolish Academy of SciencesParkowa 562‐035KórnikPoland; ^3^ Forestry Commission231 Corstorphine RoadEH12 7ATUK; ^4^ Institute of Evolutionary BiologyThe University of EdinburghAshworth BuildingCharlotte Auerbach Road, King's BuildingsEdinburghEH9 3JFUK; ^5^ Forest ResearchNorthern Research StationRoslinMidlothianEH25 9SYUK

**Keywords:** adaptation, *Dothistroma septosporum*, evolvability, heritability, Scots pine, susceptibility

## Abstract

The threat from pests and pathogens to native and commercially planted forest trees is unprecedented and expected to increase under climate change. The degree to which forests respond to threats from pathogens depends on their adaptive capacity, which is determined largely by genetically controlled variation in susceptibility of the individual trees within them and the heritability and evolvability of this trait. The most significant current threat to the economically and ecologically important species Scots pine (*Pinus sylvestris*) is dothistroma needle blight (DNB), caused by the foliar pathogen *Dothistroma septosporum*. A progeny‐population trial of 4‐year‐old Scots pine trees, comprising six populations from native Caledonian pinewoods each with three to five families in seven blocks, was artificially inoculated using a single isolate of *D. septosporum*. Susceptibility to *D. septosporum*, assessed as the percentage of non‐green needles, was measured regularly over a period of 61 days following inoculation, during which plants were maintained in conditions ideal for DNB development (warm; high humidity; high leaf wetness). There were significant differences in susceptibility to *D. septosporum* among families indicating that variation in this trait is heritable, with high estimates of narrow‐sense heritability (0.38–0.75) and evolvability (genetic coefficient of variation, 23.47). It is concluded that native Scots pine populations contain sufficient genetic diversity to evolve lower susceptibility to *D. septosporum* through natural selection in response to increased prevalence of this pathogen.

## Introduction

Forests currently face multiple threats from native and invasive pests and pathogens as well as fragmentation and climate change. These threats may have an impact individually or in combination on health, fitness and long‐term survival of trees. For example, susceptibility to threats such as native pests and pathogens may increase when trees are stressed following perturbation (Schoeneweiss, 1975). Despite long lifespans and generation times that combine to make forests particularly vulnerable to rapid change (Lindner *et al*., 2010), adaptation in tree populations can be fast (Jump *et al*., 2006), particularly where the selection pressure is high (Kremer *et al*., 2012). Resilience of forests to perturbation, such as an increase in disease, requires resistance and adaptive capacity (Lindner *et al*., 2010). The latter relies in turn on genetic and phenotypic diversity in order to buffer populations against change in the short term, and enable them to adapt to it in the long term.

Of critical importance in determining the impact of disease on forest resilience is not only phenotypic variation but also the heritability of traits that confer low susceptibility to disease (Telford *et al*., 2015). Variation in these traits must be heritable if natural populations are to adapt to change or for the trait to be incorporated into breeding programmes (McKinney *et al*., 2011). Populations with the adaptive capacity to respond to pathogens are better able to survive: they are likely to be genetically diverse with large effective population sizes and, crucially, experience no disruption to generational turnover as this is likely to be the most significant barrier to adaptive change (Cavers & Cottrell, 2015). At a population level, diversity in the expression of adaptive traits provides a measure of the amount of intraspecific adaptive genetic variation and also gives an indication of the evolvability of the trait: traits that are determined by genes containing high levels of genetic variation have a greater potential to evolve than those that are under the control of genes that have a very narrow genetic base (Houle, 1992).

Dothistroma needle blight (DNB), caused primarily by *Dothistroma septosporum*, is one of the most important diseases of pine worldwide (Brown & Webber, 2008). This is due to the broad range of pines that can act as hosts and the wide geographical range across which these occur: at least 86 species of pine are potential hosts in more than 60 countries (Watt *et al*., 2009) in every continent except Antarctica. Severity of symptoms varies widely and ranges from loss of needles and reduction in growth rate through to tree mortality. Until recently, Scots pine (*Pinus sylvestris*) was believed to be relatively resistant to *D. septosporum* (Lang & Karadžic, 1987), but the prevalence of this pathogen in plantations and natural woodlands has increased substantially in Europe over the past two decades (Boroń *et al*., 2016). In Britain, infection of Scots pine plantations and of native (Caledonian) pinewood fragments in Scotland has been reported (Brown *et al*., 2012) following recent extensive surveys.

Concern regarding the impact of DNB is focused on its financial consequences within commercial pine forests and on its conservation implications in Caledonian pinewoods. In Britain, Scots pine is highly valued both economically as an important plantation timber species and ecologically as the only native pine and the key constituent of the iconic Caledonian pinewoods (Salmela *et al*., 2010). Populations of Caledonian Scots pine are highly fragmented and have been reduced to around 1% of their maximum distribution (Kinloch *et al*., 1986). Despite this, populations retain high levels of selectively neutral variation and exhibit little or no differentiation for these markers (Kinloch *et al*., 1986; Wachowiak *et al*., 2011, 2013), which indicates that the fragments remain connected by gene flow and experience its homogenizing effects. Studies from common garden trials, in contrast, report significant genetic differentiation related to site of origin for adaptive traits including timing of growth initiation, response to seasonal temperatures and timing of bud flush (Salmela *et al*., 2011, 2013).

There is known to be a large amount of intraspecific variation in susceptibility to *D. septosporum* in Scots pine (Fraser *et al*., 2015, 2016), and high neutral genetic diversity in the pathogen (Mullett, 2014), which are potential indicators of an endemic pathosystem (Ennos, 2015). Despite this, the pathogen is assumed to be exotic to Britain due to a surge in reports of cases in the 1990s (Brown & Webber, 2008).

Recent work by Fraser *et al*. (2015, 2016) has found evidence for significant differences among Caledonian pinewood populations for susceptibility to *D. septosporum* following both artificial and natural inoculations. However, the absence of family structure in the design of their experiments did not allow for estimates of heritability or evolvability of this trait to be made. Indeed, heritability has not yet been estimated for traits related to susceptibility to any pathogens in Caledonian Scots pine, despite the potential economic and ecological impact. An understanding of how populations of Caledonian pine are likely to respond to *D. septosporum*, and whether they possess sufficient capacity to adapt, can help to develop science‐based management policy for the conservation of the species in this important marginal native habitat.

One of the simplest and most sensitive techniques for assessing levels of genetic variation in susceptibility within and among host populations is the artificial inoculation of a common garden progeny‐population trial (Kabir *et al*., 2013). Artificial inoculation with a single or a limited number of pathotypes (defined as individual variants of a pathogen with a unique genetic or phenotypic signature) in controlled conditions conducive to disease development allows variation in the environment to be minimized, and for quantitative genetic variation in susceptibility among populations of trees to be detected. The comparison of families within populations furthermore allows estimates of heritability and evolvability to be made.

This study aimed to assess the potential of native Caledonian Scots pine to adapt to DNB by estimating the extent of variation in susceptibility to *D. septosporum* (measured as the severity of DNB symptoms) among and within six populations from contrasting sites of origin. This was achieved by measuring the difference in response to artificial inoculation by *D. septosporum* among populations and families grown in a common environment. Where there was significant variation among families in susceptibility to *D. septosporum*, the proportion of phenotypic variation that was heritable and caused by genetically controlled mechanisms was estimated. To place these values in context, the heritability and evolvability of three morphological traits were also measured and contrasted with estimates for susceptibility to *D. septosporum*.

## Materials and methods

### Source material

#### Scots pine

Six native Scots pine forests (Black Wood of Rannoch, BW; Glen Affric, GA; Glen Loy, GL; Glen Tanar, GT; Rothiemurcus, RD; Shieldaig, SD) were selected for this study (Table 1). At each site, five open‐pollinated mother trees, growing at least 100 m apart, were selected for cone collection (*c*. 20 cones per tree) in February and March 2010.

**Table 1:** Collection and geographic data for the site of origin of Scots pine populations and families

Population	Family	*n* a	Latitude^b^	Longitude	Altitude (m a.s.l.)
Black Wood of Rannoch (BW)	1	7	56.6724	−4.32469	310
	3	7	56.6735	−4.33056	277
	5	6	56.6754	−4.32131	275
	6	7	56.6713	−4.32039	325
	7	7	56.6709	−4.31831	281
Glen Affric (GA)	1	7	57.2540	−5.02025	261
	2	7	57.2529	−5.02178	280
	3	7	57.2521	−5.02383	292
	4	7	57.2548	−5.01778	257
	6	7	57.2562	−5.01436	274
Glen Loy (GL)	1	7	56.9099	−5.12164	144
	2	7	56.9088	−5.12122	178
	3	4	56.9076	−5.12206	217
	4	7	56.9073	−5.12264	230
	5	7	56.9067	−5.12106	233
Glen Tanar (GT)	2	7	57.0258	−2.93156	310
	3	6	57.0259	−2.93011	303
	5	7	57.0259	−2.92764	285
	6	7	57.0262	−2.92503	281
	7	7	57.0280	−2.91917	275
Rothiemurcus (RM)	1	7	57.1653	−3.78906	266
	2	7	57.1660	−3.78983	262
	3^c^	14	57.1667	−3.79142	260
	4	7	57.1675	−3.79103	261
	6	7	57.1678	−3.79372	259
Shieldaig (SD)	1	7	57.5016	−5.62378	64
	5	7	57.5032	−5.62836	61
	6	7	57.5035	−5.62922	57

a *n*: number of individuals per family in the trial.

b Geographic data (latitude, longitude, altitude) were obtained during seed collection using a hand‐held GPS.

c One tree per block was inoculated (*n *=* *7), one tree per block was a negative control (*n *=* *7): total 14 trees.

All subsequent work was undertaken at the Centre for Ecology and Hydrology in Midlothian, UK (55.8612°N, 3.2078°E). Seeds were extracted from cones and germinated in May 2010 in trays of John Innes seed compost topped with sand. Families comprised 10–25 seedlings (half‐ or full‐siblings). After the first whorl of needles had emerged, individual seedlings were transferred to 11 × 11 × 12 cm pots containing a 3:1 ratio of John Innes compost (no. 3) to sand. Trees were grown in a randomized block design (one representative of each family per block up to a maximum of 25 blocks) in an unheated glasshouse for 4 years prior to the experiment. Pots were placed on capillary matting and were watered at regular intervals to field capacity. A liquid insecticide (Ultimate Bug Killer; Bayer) was applied by spray to all trees when aphids were prevalent. Prior to artificial inoculation, all dead needles were removed from the trees to prevent confusion with symptoms of DNB. The vast majority of dead needles were from previous years’ growth (2012 and earlier). Two morphological characters, height and number of branches, were also measured prior to inoculation to assess whether particular attributes of tree architecture were associated with susceptibility to infection. The total number of needles per tree was also measured post‐inoculation. The heritability and evolvability values of these morphological traits were used to set those for susceptibility to *D. septosporum* into context.

#### *Dothistroma septosporum* conidial suspension

A *D. septosporum* conidial suspension was prepared and used as described by Kabir *et al*. (2013), except for the following minor modifications: a concentration of 2.4 × 10^6^ spores mL^−1^ was used, and trees were not individually covered following inoculation. Inoculum was prepared from a single isolate, collected in May 2013, from a Scots pine in Midlothian, (55.8488°N, 3.2278°E). Germination of the conidial suspension was verified on 1.5% water agar plates: over 95% germination was observed after 24 h.

### Experimental design

A single representative plant from every family (five families in each of six populations, except population SD which comprised only three families: total 28 families) was included in each of seven randomized blocks (this was a subsample of the original randomized trial described above). Also included in each block were one negative and one positive control. The negative control was a Scots pine from family 3 of population RM (RM3) treated with deionized water instead of *D. septosporum* conidial suspension, to check whether symptoms observed in inoculated trees were the result of inoculation with *D. septosporum*, subsequent infection by inoculated plants in the chamber, symptom development due to pathogens already present in the needles (and therefore present in all trees including controls) or conditions within the chambers leading to trees becoming stressed. The positive control comprised a species known to be susceptible (Woods *et al*., 2005), Alaskan lodgepole pine (*Pinus contorta*, 2 years old, raised at Newton Nursery, Morayshire, UK) and was used to check whether the inoculant was viable. Gaps, due to insufficient seedlings within a family (*n *=* *5), were filled with trees from the same population, but results from these trees were not included in the analysis.

### Artificial inoculation

Seven chambers were constructed to house each of the seven blocks of trees. Chambers comprised a wooden frame measuring 1.2 × 1 × 1 m that was covered on the sides and top with transparent plastic sheeting. Chambers were placed on raised benching within the glasshouse. A pipe, with a misting attachment and connected to mains water, was inserted through the top of each chamber. Watering was set to 2 min h^−1^ for the first 72 h, reduced to 1 min h^−1^ between 08.00–16.00 h for the next 3 weeks, and to 1 min three times a day for the remainder of the trial. Temperature and humidity measurements within each chamber were taken hourly by a Tinytag data logger (Gemini). Glasshouse shading was applied to reduce temperatures. Mean day and night temperatures were 21.90 ± 0.07 °C and 15.36 ± 0.03 °C, respectively, in all chambers. Mean relative humidity was >99% in each chamber. Lighting was ambient throughout the experiment. Each tree was inoculated on a single occasion in February 2014 with the *D. septosporum* conidial spore suspension described above, applied using a hand‐held atomizer until large droplets formed on the needles. The trees were sprayed individually in a separate inoculation chamber, after which they were returned to the trial chambers and left to dry for at least 30 min before the misting schedule began.

### Infection assessments

Any needle that was not completely green was henceforth considered to exhibit symptoms of DNB and observations based on this definition are used to discuss infection of needles with *D. septosporum* and susceptibility of trees to *D. septosporum*. DNB severity is defined as the percentage of needles with symptoms consistent with DNB (needles with lesions and necrotic needles, i.e. all those which are not entirely green).

DNB severity was estimated visually and non‐destructively in 5% increments (as percentage needles not green, where 1% infection is equivalent to negligible symptoms). To follow the time course of infection, assessments were made at regular periods during the experiment (7, 14, 21, 28, 35, 42, 48 and 61 days post‐inoculation (dpi)). At the end of the trial, 61 dpi, needles were destructively harvested from all trees and stored at −80 °C prior to detailed assessment. At harvest these needles were separated into two age classes: current (2013) needles and previous year needles, where the latter includes all needles not in the current age class (≤2012 needles). Needles that were produced as a result of the bud burst that occurred during the experiment (2014 needles) were removed and not included in the assessment. There were two categories in each age class: needles without symptoms (entirely green needles) and needles with symptoms (needles with lesions and necrotic needles). All needles within each category were counted. All data discussed hereafter are from current age class needles only, except for positive controls and in weekly assessments of estimated infection, where all needles were included. Therefore, values for DNB severity have been calculated for each Scots pine in the trial based on the percentage of current year needles observed with symptoms consistent with DNB. Current year needles were prioritized because the majority of previous year needles in all trees became necrotic during the course of the experiment (data not shown). If previous year needles were included in assessments, trees that had a large proportion of previous year needles removed prior to the commencement of the experiment may therefore appear to have a lower susceptibility to *D. septosporum* than trees with few previous year needles removed. Previous year needles were included in assessments of DNB severity in the positive control trees as there was a limited number of total needles (due to the younger age of these trees). These trees were also primarily assessed to indicate whether the inoculation had been successful and susceptibility of these trees is not directly compared to inoculated Scots pine.

DNB severity is reported as both ‘estimated’ (from visual, non‐destructive assessments at eight time points during the experiment) and ‘actual’ (from a final detailed, destructive assessment). It was observed towards the end of the experiment that necrotic needles were dropping from inoculated trees and could therefore not be included in the final ‘actual’ or later ‘estimated’ DNB severity scores. An estimate of the percentage of necrotic needles that were dropped by inoculated trees during the experiment is obtained from the mean difference in total number of needles between the treated and negative control plants of RM3 (individuals of which were either inoculated or sprayed with water) and measures the estimated loss of needles within the inoculated trees in this family. ‘Inferred total’ DNB severity, defined as the ‘actual’ DNB severity plus the estimated percentage of necrotic needles dropped during the experiment, is also reported for RM3.

### Statistical analysis

Statistical analyses were performed using minitab v. 17. To test for significant differences in DNB severity among families, populations and blocks, nested analysis of variance (anova) tests were performed with population as a fixed effect, and families nested within population and block as random effects (excluding gap trees and positive and negative controls). In those cases in which residuals were not normally distributed, data were log transformed. To analyse the effect of treatment within a single family (RM3), anova was performed for DNB severity with treatment and block as fixed and random effects, respectively, and height as a covariate. An additional test to assess whether treatment may have led to a significant loss in the number of needles (in order to identify whether estimates of DNB severity using remaining needles may be underestimates) was performed with the same fixed and random effects and covariate as above.

Narrow‐sense heritability (*h* ^2^), the total phenotypic variance explained by additive genetic effects, was estimated using among family (V_fam_), block (V_block_) and residual (V_res_) variance from data pooled across all populations as follows:

$$h^{2}=\frac{V_{A}}{V_{P}}=\frac{RV_{\mathrm{fam}}}{V_{\mathrm{fam}}+V_{\mathrm{block}}+V_{\mathrm{res}}}$$

where V_A_ is additive genetic variance and V_P_ is phenotypic variance. Due to the uncertainty of the ratio of full‐ to half‐siblings in each family, narrow‐sense heritability estimates were calculated for three relatedness (*R*) scenarios: trees within a family are all half‐siblings (i.e. only share a ‘mother’); trees within a family are 50% full‐ and 50% half‐siblings; and trees within a family are all full‐siblings. For each of these scenarios, *R* is equal to 4, 3 and 2, respectively. Standard errors (SE) for heritability (*h* ^2^) estimates were calculated as follows following the method described by Vissher (1998):

$${\mathrm{SE}}_{h^{2}}=R\sqrt{\frac{2{\left(1-\frac{h^{2}}{R}\right)}^{2}{\left[1+\left(s-1\right)\frac{h^{2}}{R}\right]}^{2}}{s(s-1)(f-1)}}$$

where *R* is the relatedness of trees within families as previously described, *s* is the mean number of offspring per family and *f* is the mean number of families. The genetic coefficient of variation (CV_A_), a standardized measure of variation normalized by the trait mean, provides a measure of the evolvability of a trait (Houle, 1992). It was estimated for each trait as:

$${\mathrm{CV}}_{A}=\frac{\sqrt{V_{A}}}{\mu_{\mathrm{trait}}}\times 100$$

where *μ*_trait_ is the mean of the trait of interest.

Correlations between ‘estimated’ DNB severity and ‘actual’ DNB severity for each tree (DNB severity estimated on all needles, current and previous, for all trees within all blocks) were performed to assess the strength of their relationships and to compare assessment techniques.

## Results

### Effect of treatment

#### Positive and negative controls

**Figure 1:**
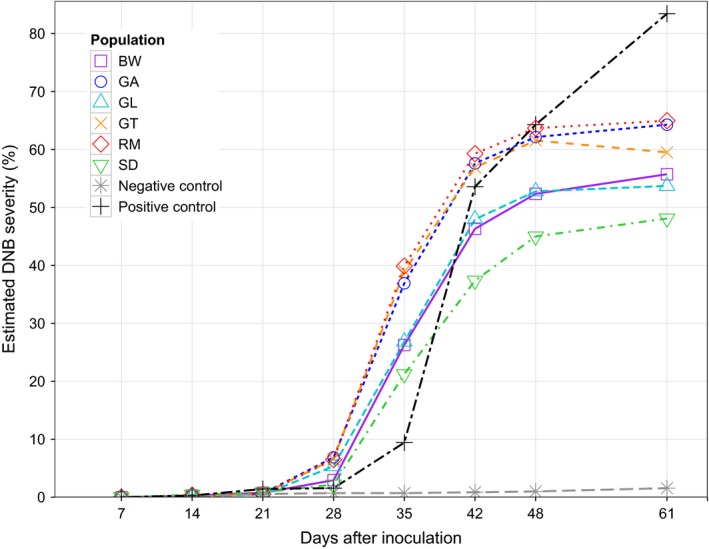
Temporal increase in mean estimated percentage dothistroma needle blight (DNB) severity for each Scots pine population and controls. Positive controls comprised a species known to be susceptible, Alaskan lodgepole pine, to check the inoculum was viable. Negative controls were Scots pine trees from family 3 of population RM (RM3) treated with deionized water instead of *Dothistroma septosporum* conidial suspension to check symptoms observed were due to inoculation. Populations: BW, Black Wood of Rannoch; GA, Glen Affric; GL, Glen Loy; GT, Glen Tanar; RM, Rothiemurcus; SD, Shieldaig.

Symptoms consistent with DNB (necrotic needles and needles with lesions) were observed at a very low level in negative control trees throughout the experiment (Fig. 1). DNB symptoms remained low in negative control trees at the end of the experiment (non‐inoculated RM3 mean ‘actual’ DNB severity, 2.3 ± 0.6%; Table S1) and were significantly less than observed in inoculated trees of the same family (inoculated RM3 mean ‘actual’ DNB severity, 51.1 ± 5.2; Table S1). DNB severity values higher than those recorded for negative control trees are therefore attributed to the effects of inoculation and are used to discuss the relative susceptibility of trees to *D. septosporum*. Symptoms consistent with DNB were observed from 14 dpi in positive control trees (Fig. 1) and had reached very high levels at the end of the experiment (lodgepole pine mean ‘actual’ DNB severity, 84.3 ± 3.5%; Table S1).

The effect of treatment (inoculation) on DNB severity was assessed by comparing the same family within the same population (RM3), where one group was inoculated (*n *=* *7), and one group was a negative non‐inoculated control (*n *=* *7). Inoculated trees had higher mean proportions of needles with symptoms (mean 48.7% greater DNB severity) and fewer needles (mean 32.8% reduction) than negative controls. The effect of treatment on DNB severity was significant (anova *F*_(1,5)_ **=* *69.68, *P *<* *0.001) as was the effect of treatment on total number of needles remaining (anova *F*_(1,5)_ **=* *6.63, *P *=* *0.05). There was a 12.9% increase in the ‘inferred total’ DNB severity as compared to the ‘actual’ DNB severity in inoculated RM3 trees (means: ‘actual’, 51.1%;‘inferred total’, 57.6%) if an estimation of the percentage of current year needles which dropped (and were therefore not counted) during the experiment is allowed for.

#### Inoculated Scots pine

**Figure 2:**
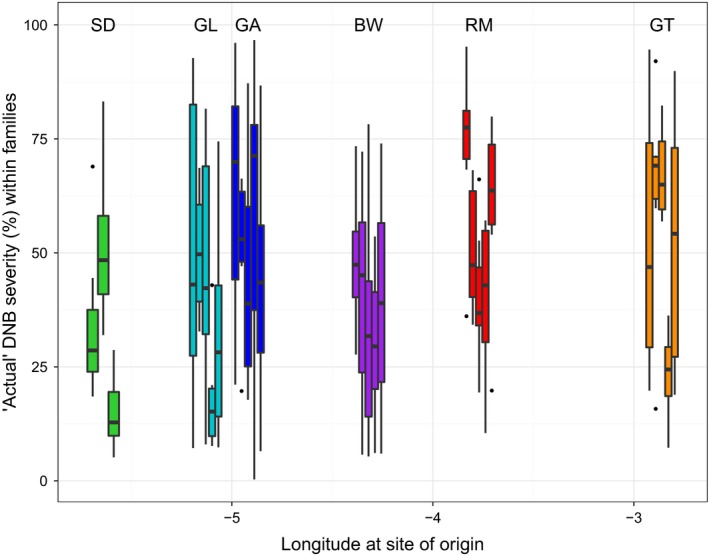
Box and whisker plot of dothistroma needle blight (DNB) severity for each Scots pine family within each population, ordered by longitude. Population codes: BW, Black Wood of Rannoch; GA, Glen Affric; GL, Glen Loy; GT, Glen Tanar; RM, Rothiemurcus; SD, Shieldaig. Individual boxes represent one family. Solid black lines indicate the median DNB severity. The bottom and top of boxes indicate the first and third quartile. The upper and lower whiskers extend to the highest and lowest values within 1.5 times the interquartile range. Individual points indicate outliers.

Symptoms consistent with DNB were observed in all trees and DNB severity was normally distributed across all trees in the trial. DNB severity at the end of the experiment (‘actual’) for individual trees ranged from 0.3% to 96.7% (mean 45.5 ± 1.8%; Table S1). Individuals within families differed in their susceptibility to *D. septosporum* and some families showed greater variation than others in this trait (Fig. 2; Table S1). DNB severity ranged (i.e. the difference between the lowest and highest percentage of needles with symptoms for individuals within each family) from 23.5% (SD5) to 96.3% (GA4). Almost 8% of trees maintained relatively low susceptibility to *D. septosporum* (<10% needles with symptoms) while nearly 4% of trees were severely affected (>90% needles with symptoms).

### ‘Estimated’ DNB severity: time course of infection (visual, non‐destructive assessments)

Symptoms of DNB were first recorded at 14 dpi, with large increases in incidence and severity at each assessment until 48 dpi when symptom development appeared to plateau (Fig. 1). It was observed that needles were dropping towards the end of the experiment. This may have affected the final assessment: DNB severity in 69% of trees either reduced or did not change between 48 and 61 dpi.

There were significant differences (Table 2) in ‘estimated’ DNB severity among families at five of the eight dates when visual assessments were performed (28, 35, 42, 48 and 61 dpi). In addition, there were significant differences among populations (anova *F*_(5,22)_ **=* *3.31, *P *<* *0.05; Table 2) at 21 dpi. There were significant differences among blocks from 28 dpi until the end of the experiment (Table 2).

**Table 2:** Adjusted mean sum of squares (MS) from anova of dothistroma needle blight (DNB) severity from each ‘estimated’ visual assessment (at 7, 14, 21, 28, 35, 42, 48 and 61 days post‐inoculation) and the final ‘actual’ destructive assessment (at 61 days post‐inoculation)

Assessment type	Days post‐inoculation	Adjusted MS^a^			
		Block	Population	Family (population)	Error
Estimated	7	NA^b^	NA	NA	NA
	14	0.3	0.4	0.2	0.2
	21	0.1	0.7*	0.2	0.2
	28	109.3*	120.8	99.5**	41.4
	35	3038.5***	1791.6	1048.2*	564.5
	42	1462.8*	1975.1	1239.2**	611.6
	48	1372.0*	1481.9	1048.9*	596.5
	61	1749.6**	1108.7	1019.7*	530.5
Actual	61	1562.0**	2054.1	1225.2***	449.7

Significance values are indicated by asterisks (*, *P *<* *0.05; **, *P *<* *0.01; ***, *P *<* *0.001).

a Degrees of freedom: block* *=* *6, population* *=* *5, families (nested within population)* *=* *22, error* *=* *157.

b NA, not available. DNB severity for all trees at day 7 was 0% and the anova was therefore not possible.

### ‘Actual’ DNB severity (detailed, destructive assessment)

There was a large amount of variation in susceptibility to *D. septosporum* within and among populations of Scots pine. DNB severity was significantly different among families within populations (anova *F*_(22,157)_ **=* *2.72, *P *<* *0.001; Table 2; Fig. 2) but not among populations (anova *F*_(5,22)_ **=* *1.68, *P *=* *0.181). Estimated narrow‐sense heritability (*h* ^2^) of the trait (DNB severity) was high and ranged from 0.38 to 0.75 depending on the assumptions made regarding relatedness of trees within families (Table 3).

**Table 3:** Narrow‐sense heritability estimates (*h* ^*2*^), their associated standard errors (SE) and genetic coefficient of variation (CV_A_) for morphological traits and dothistroma needle blight (DNB) severity for inoculated Scots pine

Trait	*h* ^*2*^ ± SE^a^			CV_A_	Total variance due to^b^	
	*R *=* *2	*R* = 3	*R* = 4		Family (%)	Block (%)
Height (mm)	0.81 ± 0.47	1.22 ± 0.46	1.62 ± 0.73	12.48	40.59	1.64
No. needles	0.59 ± 0.45	0.89 ± 0.50	1.18 ± 0.86	27.61	29.17	0.00
No. branches (log)	0.53 ± 0.44	0.79 ± 0.53	1.05 ± 0.90	12.35	25.87	0.00
DNB severity (%)	0.38 ± 0.40	0.57 ± 0.67	0.75 ± 0.99	23.47	18.86	6.77

a Three relatedness scenarios are given for narrow‐sense heritability estimates: trees within families are all full‐siblings (*R* = 2); trees within families are 50% half‐ and 50% full‐siblings (*R* = 3); and trees within families are all half‐siblings (*R* = 4).

b The percentage of total variance in each trait that is due to family and block are detailed. Populations have been pooled for all estimates.

The proportion of variation in DNB severity due to family was relatively high (18.86%) with a much lower proportion of variance due to block (6.77%). Evolvability (genetic coefficient of variation) of DNB severity (CV_A_ **=* *23.47) was high (Table 3).

DNB severity estimated at 61 dpi by visual inspection was highly correlated with ‘actual’ DNB severity obtained by destructive sampling at the end of the trial *r *=* *0.88; Fig. S1). Of all trees assessed, 28% of the values for ‘estimated’ DNB severity were within 10% of those for ‘actual’ DNB severity and 49% were within 20%. Across all trees, mean ‘estimated’ DNB severity was 14% higher than the mean ‘actual’ DNB severity.

### Morphological traits

There was significant variation among families in all measured morphological traits: height (anova *F*_(22,157)_ **=* *5.78, *P *<* *0.001); total number of branches (anova *F*_(22,157)_ **=* *3.37, *P *<* *0.001); total number of needles (anova *F*_(22,157)_ **=* *3.8, *P *<* *0.001). There were no significant differences among populations for any traits.

Estimates of the proportion of variation due to family were higher for morphological traits (25.87–40.59%; Table 3) than for DNB severity (18.86%). Evolvability of morphological traits (CV_A_ 12.35–27.61) was comparable with DNB severity (CV_A_ **=* *23.47; Table 3).

DNB severity and morphological traits were highly correlated (data not shown; taller trees with many, shorter needles and branches were less susceptible to *D. septosporum* than shorter trees with fewer, longer needles and branches). However, the genetic associations between these traits cannot be tested for significance by correlation between family means due to the low number of families within each population.

## Discussion

The threat of exotic and indigenous pathogens to forest trees is of major concern to foresters and conservationists as well as to wider society for whom forest trees are an important source of recreation and beauty. Quantitative variation in the response of trees to pathogens is a key indicator of a population's ability to adapt to threats in the long term (Ennos, 2015). Durability is also expected to be greater in quantitative (as opposed to qualitative) traits that are controlled by multiple genes (Lindhout, 2002). The extent and speed with which populations are able to adapt also depends on the heritability of quantitative traits such as susceptibility to pathogens (McKinney *et al*., 2011). The findings from this study provide evidence that there is significant quantitative variation among native Scots pine families in their response to inoculation with *D. septosporum*, and that a significant proportion of this variation in response is heritable. The lack of significant differences among populations for susceptibility to *D. septosporum* is in contrast to the reported findings of Fraser *et al*. (2015), although they used different Scots pine populations and their trees were more than likely inoculated with different pathotypes: both factors may have contributed to variation in the host response. This study has furthermore found evidence that evolvability, the potential of an organism to evolve in the future, for this trait is very high in Caledonian Scots pine populations.

The levels of variation (0.3–96.7% DNB severity) and the conservative estimate of heritability in this trait (*h* ^2^ **=* *0.38) are similar to those observed in *Fraxinus excelsior* in response to *Hymenoscyphus fraxineus*, which has a very large range (0–80%) of variation in damage (McKinney *et al*., 2011) and high (0.37–0.52) levels of narrow‐sense heritability (Kjaer *et al*., 2012). Levels of variation in Scots pine were also similar to those previously observed in radiata pine in response to *D. septosporum*, although estimated heritability in Scots pine was generally higher (Chambers *et al*., 2000; Devey *et al*., 2004). However, it must be acknowledged that the heritability estimates reported here reflect inoculation with a single isolate under artificial and controlled conditions, and lower estimates would be expected if this experiment had been replicated in a natural environment. Upper estimates of heritability were above 1 for all morphological traits, suggesting that the assumption that all trees within families were half‐sibs was incorrect. It is also possible that maternal effects acted on these traits leading to an overestimation of heritability (Roach & Wulff, 1987). Standard errors were also very high for both DNB severity and morphological traits, but this was not unexpected given the relatively low sample size.

As has previously been reported, Scots pine trees that are taller tend to show lower susceptibility to *D. septosporum* (Fraser *et al*., 2015). This study has found evidence that these traits are heritable, but it has not enabled testing of whether these traits are genetically correlated with one another: a similar experimental design using greater numbers of families per population would be required to estimate genetic correlations between traits. If a genetic correlation could be established between morphological and susceptibility traits, response to *D. septosporum* could be predicted by tree breeders based on physical characteristics. Kennedy *et al*. (2014) reported a strong genetic correlation between DBH (diameter at breast height) and susceptibility to *D. septosporum* in an even‐aged trial of radiata pine in New Zealand and advocated selecting for stem diameter following severe DNB in breeding populations. Fraser *et al*. (2015) have proposed three possible explanations for apparent greater susceptibility to *D. septosporum* in shorter compared to taller trees of the same age, namely: shorter trees are less vigorous; the greater proximity of their needles provides a microclimate that is more optimal for infection; they are exposed to greater secondary infection pressure from water‐displaced conidia originating from taller trees.

In contrast to previous artificial inoculation studies (Kabir *et al*., 2013; Fraser *et al*., 2015), needles were not checked for *D. septosporum* acervuli in this study. This was due to time constraints and to the fact that needles with symptoms do not always have erumpent acervuli (Millberg *et al*., 2015). A possible explanation for the lack of acervuli in needles with symptoms is that these needles are infected with *D. septosporum* but are at a different stage of infection (i.e. acervuli have yet to erupt from the epidermis). Another possibility is that host defence mechanisms are able to restrict sporulation but not the development of necrosis on the needles. Alternatively, infection by *D. septosporum* may have led to a decline in the overall health of the tree, which may have led to uninfected needles becoming necrotic or more vulnerable to other pathogens (Schoeneweiss, 1975). Whatever the underlying reason, in all cases the assumption that these needles are indicative of susceptibility to *D. septosporum* was justified as nearly all needles with symptoms could be attributed to infection by *D. septosporum* and not, for example, to the conditions within the inoculation chambers. This was evidenced by comparison of inoculated trees with negative controls, where the negative controls retained only a very low proportion of needles with symptoms throughout the experiment. That all negative control trees developed very low levels of symptoms may either indicate that trees were infected prior to the start of the experiment, or alternatively, there may have been some degree of cross‐infection within the chambers during the experiment. It is important to note that variation in acervuli production (either their presence or frequency) within or among populations or families may be due to the effect of host defence mechanisms. Two individuals with ostensibly the same susceptibility to *D. septosporum* may differ in their long‐term susceptibility if the pathogen is able to maintain a very high inoculum load (i.e. the number of acervuli producing spores) on one but not another. This is an area that would benefit from further research in the future.

The use of two assessment techniques, ‘estimated’ (visual estimations of susceptibility) and ‘actual’ (destructive counting of needles with symptoms), allows these different approaches to be compared. Although ‘estimated’ susceptibility measurements were generally higher than ‘actual’ susceptibility, the close correlation between them and the flexibility that ‘estimated’ assessments afford highlights their value. Given that DNB severity among families was significantly different from 28 dpi through to the final assessment, it is clear that variation in this trait can be assessed even at low levels of infection.

The high estimated loss of needles from trees observed in this experiment as a result of DNB must also be considered when interpreting the results. Given the fragile hold that necrotic needles have on the stem, it is probable that many dropped during the course of the experiment and were therefore not recovered, counted or included in results. Despite losing necrotic needles towards the end of the experiment, the duration of the experiment was appropriate as it allowed symptom development to plateau before the end of the experiment. Although it was possible to estimate the extent of needle loss during the experiment for a single family, there was no data with which to estimate how the proportion of necrotic needles lost varied between individuals, families or populations, or with different levels of susceptibility. It is probable that families with comparatively low counts of needles with symptoms and low susceptibility to *D. septosporum* will have lost fewer needles than families with high counts of needles with symptoms and high susceptibility. This may mean that the difference in susceptibility to *D. septosporum* among individuals, families and populations was underestimated in this experiment. Another consideration is that needle shedding in Scots pine is a form of defence, as has been observed in *Pinus monticola* in response to *Cronartium ribicola* (McDonald & Hoff, 1971).

*Dothistroma septosporum* is generally thought to be an introduced pathogen to Britain. The increase in the prevalence of DNB has been attributed to multiple introductions of the pathogen through infected stock, an increase in planting and availability of susceptible species (Corsican (*Pinus nigra* subsp. *laricio*) and lodgepole pine) and a changing climate that is becoming more optimal for the pathogen (Brown *et al*., 2012). The extent of variation in susceptibility to *D. septosporum* within and among populations and families and the discovery that a significant proportion of this variation is heritable may indicate that native Scots pine in Scotland has been exposed to *D. septosporum* for significantly longer than has previously been assumed. This would be in line with progress of the disease in Canada where it has been suggested that *D. septosporum* is a native rather than a recently introduced species (Welsh *et al*., 2009). In British Columbia, dendrochronological records indicate lodgepole pine may have co‐existed with low levels of the pathogen for at least 180 years (Welsh *et al*., 2009) and it is only in the last 15 years that the prevalence and severity of DNB on lodgepole pine has increased dramatically and resulted in extensive damage and mortality (Woods *et al*., 2005). The increase in severity has been attributed to extensive planting of susceptible pines (predominantly lodgepole pine) and to a changing climate that favours the pathogen (Woods *et al*., 2005). If circumstances in Britain are indeed similar to those in British Columbia, the severity and impact of DNB on Scots pine may increase as the climate changes and if alternative susceptible non‐native species are introduced to timber production plantations. An understanding of the extent and speed with which native trees are likely to be able to adapt may aid in minimizing negative impacts of DNB through careful and informed management of native pinewood and tree breeding programmes.

Although this study provides evidence for the contribution of heritable adaptive genetic variation in susceptibility of Scots pine to *D. septosporum*, the conditions in this experiment were necessarily simplified as compared to natural conditions. Results obtained via natural inoculation of a field‐based trial are consequently likely to be more complex and variable (Fraser *et al*., 2016), although more of a true reflection of what happens in the wider forest. A single isolate of *D. septosporum* was used to inoculate all trees and the environment was controlled where possible to provide optimal conditions for infection. A longer‐term field‐based progeny‐population trial including a larger number of populations, where trees are subject to field inoculum under natural conditions, has also been established. This study provides an appropriate design to test whether variation in susceptibility to *D. septosporum* among populations of native Scots pine is associated with climate at their site of origin. This may furthermore indicate whether there is evidence for adaptive genetic differentiation in the trait, and whether *D. septosporum* is potentially endemic to Great Britain.

The results from this experiment offer hope for the future of native Scots pine forests: even under conditions designed to be optimal for infection, there was massive variation in susceptibility of individuals to *D. septosporum* both within and among populations. Variation in susceptibility is also likely to be durable if it is polygenically controlled. Evidence that variation in susceptibility to *D. septosporum* is heritable also suggests that evolutionary adaptation following selection for this trait is possible. Scots pine may therefore have the adaptive capacity to survive DNB. However, this relies on active management of native pinewoods, including deer and grazing management, restocking and regeneration to allow the establishment of new generations on which natural selection can operate. It also depends on careful management of plantations, both to reduce disease pressure where possible and potentially to incorporate disease resistant breeding stock. If forests are monitored and managed well, the impact of pathogens may be lessened and likelihood of the long‐term survival of the host increased. Given that native pinewoods may provide a useful source of genetically diverse breeding stock for nurseries, applying knowledge gained when studying native Scots pine to commercial forestry may also be extremely valuable.

## Supporting information


**Figure S1** Correlation of ‘estimated’ (visual, non‐destructive assessment) and ‘actual’ (detailed, destructive assessment) dothistroma needle blight (DNB) severity of every tree in the trial (except gap trees) at 61 days post‐inoculation. Positive and negative controls are included. Positive controls comprised a species known to be susceptible, Alaskan lodgepole pine, to check the inoculum was viable. Negative controls were Scots pine trees from family 3 of population RM (RM3) treated with deionized water instead of *D. septosporum* conidial suspension to check symptoms observed were due to inoculation. As all needles were included in infection assessments for estimates, both current (2013) and previous (≤2012) age classes from the detailed assessment (‘actual’ DNB severity) are included. Points have been jittered for clarity. Correlation coefficient (*r*), significance (*P*) and degrees of freedom (d.f.) are indicated.

**Table S1** Mean and standard error (SE) values for ‘actual’ dothistroma needle blight (DNB) severity of Scots pine populations and families and positive and negative controls at the end of the experiment
