# Supplementary figures and images for: Substantial heritable variation for susceptibility to Dothistroma septosporum within populations of native British Scots pine (Pinus sylvestris)
Source: Plant Pathol. 2016 Apr 4;65(6):987–96. doi: 10.1111/ppa.12528 (PMC4984854; doi:10.1111/ppa.12528)

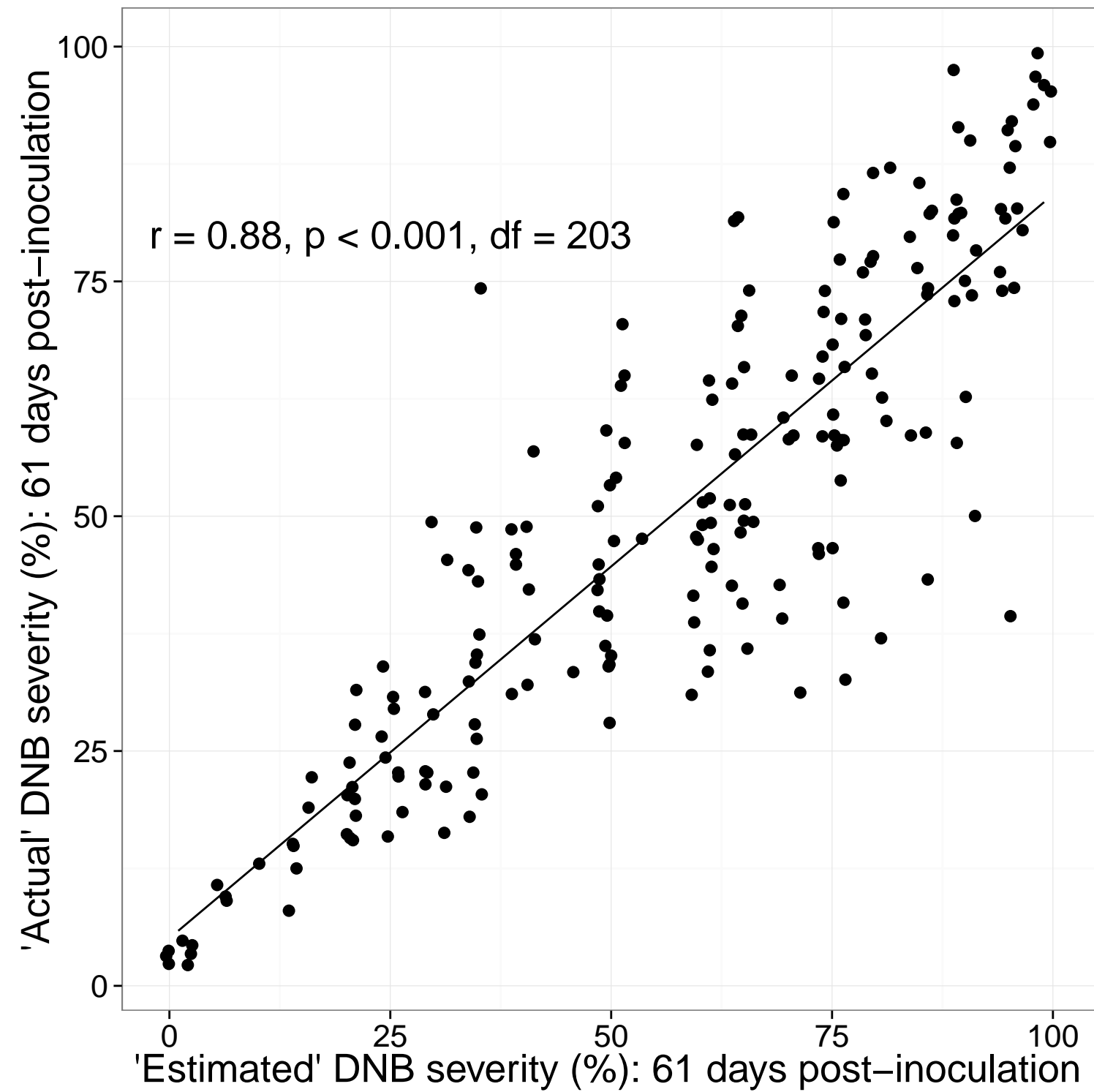

Supplement: Supplementary file 1 — Figure S1 Correlation of ‘estimated’ (visual, non‐destructive assessment) and ‘actual’ (detailed, destructive assessment) dothistroma needle blight (DNB) severity of every tree in the trial (except gap trees) at 61 days post‐inoculation. Positive and negative controls are included. Positive controls comprised a species known to be susceptible, Alaskan lodgepole pine, to check the inoculum was viable. Negative controls were Scots pine trees from family 3 of population RM (RM3) treated with deionized water instead of D. septosporum conidial suspension to check symptoms observed were due to inoculation. As all needles were included in infection assessments for estimates, both current (2013) and previous (≤2012) age classes from the detailed assessment (‘actual’ DNB severity) are included. Points have been jittered for clarity. Correlation coefficient (r), significance (P) and degrees of freedom (d.f.) are indicated. [file PPA-65-987-s001.pdf]
