# Supplementary material for: Substantial heritable variation for susceptibility to Dothistroma septosporum within populations of native British Scots pine (Pinus sylvestris)
Source: Plant Pathol. 2016 Apr 4;65(6):987–96. doi: 10.1111/ppa.12528 (PMC4984854; doi:10.1111/ppa.12528)
Supplement: Supplementary file 2 — Table S1 Mean and standard error (SE) values for ‘actual’ dothistroma needle blight (DNB) severity of Scots pine populations and families and positive and negative controls at the end of the experiment [file PPA-65-987-s002.docx]

**Table S1**. Mean and standard error (se) values for ‘actual’ DNB severity of Scots pine populations and families and positive and negative controls at the end of the experiment.

|  | N | Mean DNB severity (%) ± se |  |  | N | Mean DNB severity (%) ± se |
| --- | --- | --- | --- | --- | --- | --- |
| *Controls* | | | | | | |
| Negative | 7 | 2.34 ± 0.59 |  | Positive | 7 | 84.32 ± 3.54 |
| *Populations* | | | | | | |
| SD | 21 | 33.62 ± 4.58 |  | BW | 34 | 38.62 ± 3.65 |
| GL | 32 | 39.00 ± 4.70 |  | RM | 35 | 53.09 ± 3.49 |
| GA | 35 | 52.01 ± 4.54 |  | GT | 34 | 51.32 ± 4.45 |
| *Families* | | | | | | |
| SD1 | 7 | 15.07 ± 3.09 |  | BW1 | 7 | 40.57 ± 8.96 |
| SD5 | 7 | 51.67 ± 6.70 |  | BW3 | 7 | 48.34 ± 5.61 |
| SD6 | 7 | 34.13 ± 6.62 |  | BW5 | 6 | 33.85 ± 10.91 |
|  |  |  |  | BW6 | 7 | 30.33 ± 6.31 |
|  |  |  |  | BW7 | 7 | 39.35 ± 9.36 |
|  |  |  |  |  |  |  |
| GL1 | 7 | 47.75 ± 10.13 |  | RM1 | 7 | 60.48 ± 7.68 |
| GL2 | 7 | 17.98 ± 4.58 |  | RM2 | 7 | 40.15 ± 6.93 |
| GL3 | 4 | 50.20 ± 8.05 |  | RM3 | 7 | 51.06 ± 5.21 |
| GL4 | 7 | 51.85 ± 12.94 |  | RM4 | 7 | 40.59 ± 5.69 |
| GL5 | 7 | 31.99 ± 8.88 |  | RM6 | 7 | 73.19 ± 6.95 |
|  |  |  |  |  |  |  |
| GA1 | 7 | 44.91 ± 10.17 |  | GT2 | 7 | 52.58 ± 10.82 |
| GA2 | 7 | 51.70 ± 6.09 |  | GT3 | 6 | 62.87 ± 10.37 |
| GA3 | 7 | 62.82 ± 10.63 |  | GT5 | 7 | 67.45 ± 3.68 |
| GA4 | 7 | 57.05 ± 13.33 |  | GT6 | 7 | 23.41 ± 3.68 |
| GA6 | 7 | 43.57 ± 10.47 |  | GT7 | 7 | 51.92 ± 10.80 |

Population codes as described in Table 1. N, number of samples. Populations and families are ordered longitudinally, west to east.
